# Gelsiminum in Cough

**Published:** 1876-03

**Authors:** 


Gelsiminum in Cough.—Gelsiminum as a remedy for cough is advocated by Dr. J. R. Thompson in the *British Medical Journal.* He has administered it recently to a large number of patients suffering from a pulmonary disease (as, for example, chronic phthisis) as a cough sedative. The tincture is given in five-minim doses. When much bronchial irritation existed. he combined it with bromide of ammonia, tincture of squill, and syrup of codeia. He claims that his results show that gelseminum has a marked power in subduing cough, that it acts probably as a nervous sedative, that it is useful when other sedatives have failed, that it seldom produces any unpleasant general effect, and that the kinds of cough in which it may be administered with advantage are very varied.—*Medical Brief.*

